# Linear Combination Properties of the Phasor Space in Fluorescence Imaging

**DOI:** 10.3390/s22030999

**Published:** 2022-01-27

**Authors:** Belén Torrado, Leonel Malacrida, Suman Ranjit

**Affiliations:** 1 Laboratory for Fluorescence Dynamics, Department of Biomedical Engineering, University of California, Irvine, CA 92697, USA; btorrado@uci.edu; 2 Departamento de Fisiopatología, Hospital de Clínicas, Facultad de Medicina, Universidad de la República, Montevideo 11600, Uruguay; lmalacrida@hc.edu.uy or; 3 Advanced Bioimaging Unit, Institut Pasteur Montevideo, Universidad de la República, Montevideo 11600, Uruguay; 4 Department of Biochemistry and Molecular & Cellular Biology, and Microscopy & Imaging Shared Resources, Georgetown University, Washington, DC 20007, USA

**Keywords:** linear combination of phasor, phasor, FLIM, hyperspectral imaging, spectral phasor, fractional intensity, multiple component analysis, model-free, multidimensional phasor plot

## Abstract

The phasor approach to fluorescence lifetime imaging, and more recently hyperspectral fluorescence imaging, has increased the use of these techniques, and improved the ease and intuitiveness of the data analysis. The fit-free nature of the phasor plots increases the speed of the analysis and reduces the dimensionality, optimization of data handling and storage. The reciprocity principle between the real and imaginary space—where the phasor and the pixel that the phasor originated from are linked and can be converted from one another—has helped the expansion of this method. The phasor coordinates calculated from a pixel, where multiple fluorescent species are present, depends on the phasor positions of those components. The relative positions are governed by the linear combination properties of the phasor space. According to this principle, the phasor position of a pixel with multiple components lies inside the polygon whose vertices are occupied by the phasor positions of these individual components and the distance between the image phasor to any of the vertices is inversely proportional to the fractional intensity contribution of that component to the total fluorescence from that image pixel. The higher the fractional intensity contribution of a vertex, the closer is the resultant phasor. The linear additivity in the phasor space can be exploited to obtain the fractional intensity contribution from multiple species and quantify their contribution. This review details the various mathematical models that can be used to obtain two/three/four components from phasor space with known phasor signatures and then how to obtain both the fractional intensities and phasor positions without any prior knowledge of either, assuming they are mono-exponential in nature. We note that other than for blind components, there are no restrictions on the type of the decay or their phasor positions for linear combinations to be valid—and they are applicable to complicated fluorescence lifetime decays from components with intensity decays described by multi-exponentials.

## 1. Introduction

**Figure 1:**
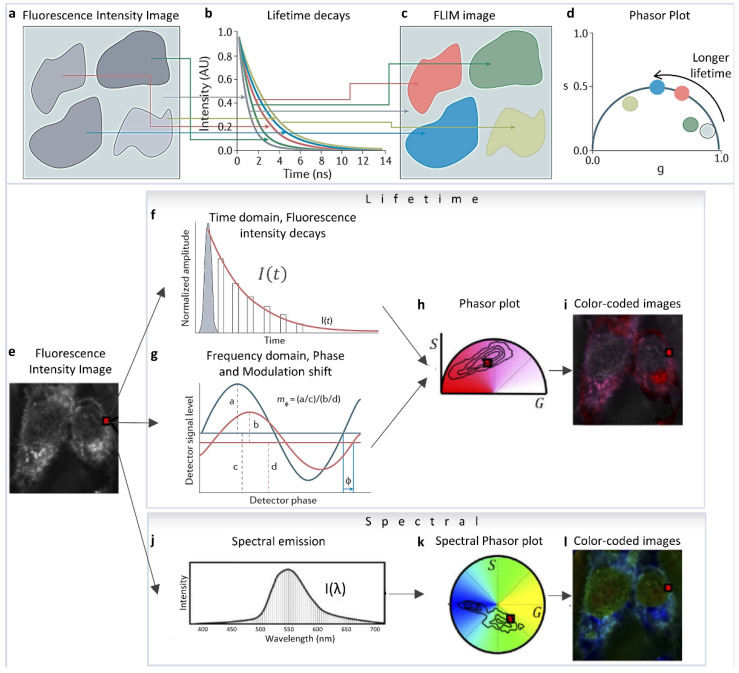
Phasor transformation. (**a**) Fluorescence lifetime imaging microscopy (FLIM) measures the fluorescence lifetime decays in different parts of the image (**b**), and color-codes areas (**c**) according to their decay times (**b**) or their phasor clusters (**d**). The resulting phasor plot (**d**) represents species that have longer fluorescence lifetimes by larger phase angles. Mono-exponential lifetimes (that is, fluorescence decays that can be fitted with a single exponential and are indicative of a single species) appear on the dark-blue semicircle (known as the ‘universal’ semicircle), whereas multi-exponential lifetimes/sum of exponential lifetimes (where the fluorescence decays require fitting with multiple exponentials, indicative of multiple fluorescent species) appear inside the semicircle. (Panels **a**–**d** adapted with permission from [27]). (**e**–**i**) Lifetime phasor transformation. (**e**) The intensity image. Lifetime data collected (at each pixel, shown with a red square) are transformed to a point in the phasor plot. Phasor points in h can be generated from Fourier transformation of lifetime decays obtained using time-correlated single-photon counting (TCSPC; **f**) or from frequency domain fluorescence lifetime imaging (**g**) using the phase shift (ϕ) (change in the x-axis) and loss of modulation (decrease in the y-axis, m) of the fluorescence signal (red) compared with the excitation signal (dark blue). In TCSPC, the decay is calculated by measuring the delay of the fluorescence signal (red) relative to the laser pulse (grey). (**i**) The phasor plot can be used to color-code the original image. (**e**,**j**–**l**) Spectral phasor transformation. The transformation is analogous to the lifetime but using spectral data. (Panels (**e**,**h**–**l**) adapted from [30]).

The phasor approach to fluorescence lifetime and hyperspectral imaging has expanded applicability and the quantitative aspect of fluorescence analysis due to its ease of implementation and its capability to simplify and handle a large amount of data [1]. The mathematics were originally formulated by Weber et al. [2], then it was first implemented by Jameson et al., for fluorescence lifetime measurements in 1984 [3], and in the early 2000s by the Gratton [4,5,6,7,8] and Clegg labs [9,10,11] in Fluorescence Lifetime Imaging Microscopy (FLIM). They showed the simplicity and easy applicability of this technique in Förster Resonance Energy Transfer (FRET) [7,12,13,14,15,16] and autofluorescence metabolic FLIM imaging [5,6,17,18,19,20,21,22,23,24]. Different areas of the fluorescence image (Figure 1a) can be identified by their difference in fluorescence lifetime decays (Figure 1b). The lifetime decays and their phasor representation (Figure 1d) can then be used to separate areas of the intensity image according to their lifetime (Figure 1c) and add information depth and dimensions to the simple fluorescence image. Phasor plots are fit-free methods for data analysis and interpretation that involves transforming fluorescence decays (for FLIM, Figure 1e–i) and spectra (for hyperspectral imaging, Figure 1j–l) to the Fourier space [1,4,25,26]. In FLIM imaging, this operation means calculating the phasor coordinates (Figure 1h) from the decay at each pixel (Figure 1a–d [27]), either from the time domain intensity decays (Figure 1f) or the frequency domain measurements (Figure 1g). Please see [28] for more details and historical perspective. This calculation results in individual decay components being additive in the Fourier space (phasor space) [29] because of the validity of vector addition properties. In both phasor FLIM or spectral phasor cases, a single pixel can have components from multiple species, and the linear addition properties allow for the separation of these components. This statement is different from the scenario wherein the components are spatially segregated in different parts of the image—as the corresponding phasor points from those different areas will be segregated in different parts of the phasor space [1]. The focus of this review is to highlight how the linear addition properties of the phasor space can be used to identify and understand changes in biological samples and how to use the phasor transformations for the quantification of various fluorescence moieties in images.

The vector addition properties of the Fourier space give rise to the linear additivity of the phasors. According to this principle, when the decay from an image pixel is a combination of the fluorescence decays of multiple species, then the position of that phasor point is going to be within the polygon, where the vertices of the polygon are occupied by the phasor points generated from the individual components [1,4,29,31]. In simplified language, this means that, for a pixel with two lifetime components, the phasor point will be along the line joining the two components. In the case of an image pixel with three lifetime components, the phasor position of that image pixel will be inside a triangle where the component phasor points are at the three vertices. The exact position of the multiple-component phasor point is determined by the fractional intensity contribution of each of the contributing components, and the distance between them is inversely proportional to the fractional intensity contribution of the corresponding contributing species. In simpler terms, this principle means that the more the fractional intensity contribution, the closer the image phasor point is to the contributing species phasor. This rule enables us to deconvolute the fractional intensity contributions of multiple contributing species using the phasor positions and the knowledge of the individual phasor components. This can be carried out for up to three components (for single frequency measurements) [17,32,33,34,35,36,37] or four components (using at least two harmonic frequencies). Blind deconvolution without a priori knowledge of the contributing phasor components is also feasible assuming the components have mono-exponential decays and multiple harmonics measurements are available.

**Figure 2:**
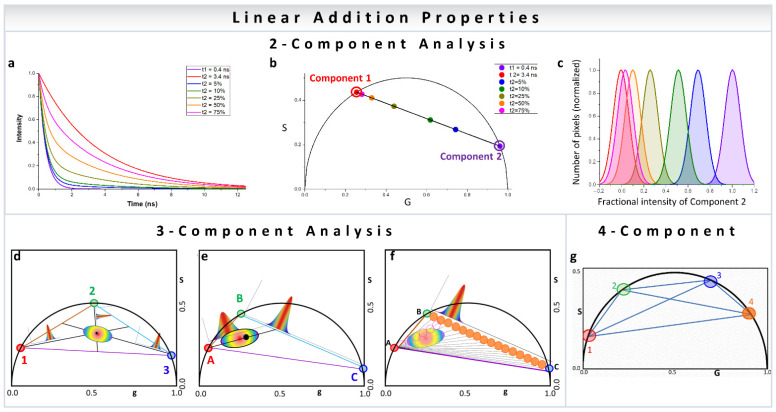
Multicomponent analysis in phasor plot. (**a**–**c**) Two-component analysis using phasor approach. (**a**) The intensity decays were calculated using the lifetime of free NADH (0.4 ns, violet) and protein-bound NADH (3.4 ns, red). Different fractions of free to bound NADH were used for calculating the decays in the middle. In increasing percentage of bound NADH—5% (blue), 10% (green), 25% (yellow-green), and 50% (orange). (**b**) The corresponding phasor positions were calculated, and they appear along the metabolic trajectory [18], connecting the positions of free (violet) and bound (red) NADH. Two-component analysis allows the distribution along the line joining the phasor positions of the individual components to be calculated, as exemplified by free and protein-bound NADH here. However, this is valid for any two original phasor components. (**d**–**f**) Three-component analysis using phasor approach. (**d**) The lines joining the individual cursor positions (red, blue and green), forming the vertices of the triangle and the phasor point whose fractional intensity contributions are being calculated (black), are extended to the opposite side of the triangle. The distances of the vertices from the black point are used for the calculation of fractional intensity contribution (the graphs). (**e**) Calculation of three components where two components are related, e.g., free and protein-bound NADH. In this case, the presence of the third component shifts the distribution towards that third component and away from metabolic trajectory. This distance can be converted to the fractional intensity of free NADH and plotted (the graph between green and blue points) or fractional intensity of third species (the graph along the line joining the red vertex and the opposite line). (**f**) Graphical analysis of three components. A cursor of a given size is scanned along point A and another point in line BC to calculate the fraction of the third component along the axis and then this process is combined for different points on BC to create the fraction of the third component. The number of points along each of these black lines are counted by scanning (orange cursors) and plotted along the coordinates of BC to calculate the fraction of free NADH. (Panels **d**–**f** adapted with permission from [32]). (**g**) Four-component analysis using phasor approach. Representation of four species with different phasors indicated by the number 1 to 4 (1, red; 2, green; 3, blue; 4, orange). In this case, the individual species are single exponentials since they are on the universal semicircle. The position of a phasor point in a single harmonic inside this quadrangle can be defined by multiple triangles. Multiple harmonics are needed to obtain unique solution for the contribution of the four components. (Panel **g** adapted with permission from [35]).

In a two-component system, wherein the resulting phasor points have two individual decay components, the position of the resulting phasor lies on the line joining the phasor positions of the individual components (Figure 2a,b). Increasing fractional intensity of one component brings the phasor cloud closer toward that component (Figure 2b,c). In a three-component system, the position is within a triangle whose vertices are the phasor positions of the individual components (Figure 2d–f) [31,32]. This approach can obviously be expanded to any number of components and polygons (Figure 2g) [35], with the only caveat being that one of the contributing species’ phasor positions cannot be along a line joining two other contributing phasor positions. The other important thing to remember is that the component deconvolution requires high precision for the calculation of the phasor position from the image which related to the signal to noise (S/N) ratio. The deconvolution calculation does not add large uncertainties in the component calculations, but the inherent S/N ratio can be carried over to the final analysis [35]. High signal to noise ratio of the image acquisition results in improved calculation of the components.

Another important aspect to note is that the mathematical calculations (published before and reproduced below) do not require the individual components to be mono-exponential (other than the blind deconvolution case). Thus, the results are truly representative of the molecular species and not the mono-exponential components, and the assumptions associated with it.

The conversion of the various phasor points to these individual contributions are the focus of this review. We show how two-, three- and four-component phasors can be converted to their individual fractional intensity contributions [32,33,35]. Then, we show how the linear additivity properties can be used for blind deconvolution of individual components, where the original phasor positions of the individual components are unknown [38]. We then delve into how other mathematical models, especially non-Euclidean geometry, can be used to obtain similar results [36,37]. These theoretical discussions are followed by discussions of FLIM and spectral data in the biological context.

There are situations wherein linear combination breaks down, often due to improper transformation of the time correlated single photon counting (TCSPC) intensity decays to the phasor space [39,40] or due to application of further restrictions, e.g., diffusion phasor. Similar non-linearity can be observed when only a part of the fluorescence spectra is transformed in the case of spectral phasors. These explanations are finally followed by the potential use of linear combinations in phasor space and a short discussion about the multidimensional phasor approach based on the reciprocity principle [1]—the ability to connect many dimensions, as spectra (for many excitation wavelengths) and lifetime (for many emission channels) can be connected for the pixel-wise analysis of same field of view.

## 2. Mathematics

### 2.1. Mathematics of Phasor and Linear Combination in FLIM

Mathematically the phasor and the linear additivity can be explained the following way [1,4,31]:

Phasor analysis of FLIM data involves Fourier transformation of the fluorescence lifetime decay curves *I*(*t*) (for time domain measurements) from each pixel. The phasor coordinates (*G, S*) are calculated using the transformations below [2]:

(1) $$G=\frac{\int_{0}^{T}\;I\left(t\right)\cos\left(n\omega t\right)\;dt}{\int_{0}^{T}\;I\left(t\right)\;dt}$$

(2) $$S=\frac{\int_{0}^{T}I\left(t\right)\sin\left(n\omega t\right)dt}{\int_{0}^{T}I\left(t\right)dt}$$

where the harmonic number *n* represents the integer number of whole cycles the trigonometric function has in the repetition period, and *ω* the angular light modulation frequency of the excitation. *ω* is equal to 2π/*T*, where *T* is the period of the laser pulses, and *I*(*t*) is the intensity decay measured from time domain measurements (Figure 1f).

In frequency domain measurements, *G* and *S* are calculated using the following transformations [2]:

(3) G=M·cos(ϕ)

(4) S=M·sin(ϕ)

where *ϕ* is the phase shift (Figure 1g) in the fluorescence signal relative to the excitation signal, defined by the delay and shift along the X direction of the fluorescence signal compared to the excitation, and *M* is the relative modulation (Figure 1g). They are related to τ (the exponential lifetime of the excited state), and *ω* (the angular light modulation frequency in radians) using following equations:

(5) tanϕ=ωτ

(6) $$Relative\;modulation=M=\frac{M_{EM}}{M_{Ex}}=\frac{a/c}{b/d}$$

A mono-exponential decay in time domain measurement can be defined as:

(7) $$I\left(t\right)=Ae^{\frac{-t}{\tau }}$$

Solving these equations define in the universal semicircle, where all the mono-exponential decays lie in the phasor plot:

(8) $$s_{i}^{2}=g_{i}-g_{i}^{2}$$

In the frequency domain, similar logic can be followed and the equation for the universal semicircle can be achieved when:

(9) M=cosϕ

In the case of multiple components and species, the intensity decays are described as the sum of exponential curves:

(10) $$I\left(t\right)=\overset{N}{\underset{1}{\sum }}A_{i}e^{\frac{-t}{\tau }}$$

Solving for the *G* and *S* coordinates of the phasor plot results in the law of linear addition, where the resulting phasor coordinates from these pixels are the sum of the phasor transforms of the pure components:

(11) $$\overset{N}{\underset{1}{\sum }}f_{i}g_{i}=G$$

(12) $$\overset{N}{\underset{1}{\sum }}f_{i}s_{i}=S$$

where *g_i_* and *s_i_* are the phasor positions of the original contributing species, and *fi* is the fractional intensity contribution to the total fluorescence of the *i*th component. A point to emphasize again is that the decays are not required to be mono-exponential. An important point to note is that the linear combination arises from the properties of Fourier space and the fact that the calculation of *G* and *S* involves division by the total intensity (Equations (1) and (2)). This normalization results in linear additivity. Please see [31] for further calculation of the two-component situation.

As mentioned above, the linear combination property of phasors allows us to convert lifetime decay information to the independent contributing species and their quantitative contribution towards the image phasor point. The contribution is originally calculated as a fractional intensity contribution, which is, of course, dependent on the quantum yield of the species. Knowledge of the quantum yield of the individual component allows for transforming the fractional intensity contribution to the molar fraction calculation.

### 2.2. Mathematics of Spectral Phasor

For more than a decade, spectral data have been available in microscopy. Instead of collecting steady-state data using discrete bandpass filters, it is possible to collect all the emitted photons using a dispersive element and a PMT-array for a step-by step acquisition for different wavelengths. Considering that the spectral imaging consists of a fixed period, it is possible to transform the data into the phasor space [25,41]. Fereidouni et al. [25] introduced the spectral phasor transformation for spectral demixing following the rules described before for fluorescence decays. The spectral phasor transformation captures the center of mass (phase, *ϕ*) and full-width-of-the-half-maximum (FWHM) (Modulation, *M*) of the spectra. The spectral phasor transformation relates to the four-quadrant phasor plot [25,42], instead of the universal circle. The real (x) and imaginary (y) components are determined as follows:

(13) $$x\;\mathrm{coordinate}=G(\lambda )=\frac{\int_{\;\lambda min}^{\;\lambda max}I\left(\lambda \right)\cos\left(\frac{2\pi n\left(\lambda -\lambda_{min}\right)}{\lambda max-\lambda min}\right)d\lambda }{\int_{\lambda min}^{\lambda max}I\left(\lambda \right)d\lambda }$$

(14) $$y\;\mathrm{coordinate}=S(\lambda )=\frac{\int_{\;\lambda min}^{\;\lambda max}I\left(\lambda \right)\sin\left(\frac{2\pi n\left(\lambda -\lambda_{min}\right)}{\lambda max-\lambda min}\right)d\lambda }{\int_{\lambda min}^{\lambda max}I\left(\lambda \right)d\lambda }$$

where *I(λ)* represents the intensity at each step of the spectrum, *n* is the number of the harmonic, and (*λ*_max_ − *λ*_min_) the spectra period in the PMT array (usually 32 parallel channels) or a range used in the step-by-step acquisition. For each pixel, the phase (ϕ) and modulation (*M*) is calculated as follows [43]:

(15) $$\phi =\arctan\left(S_{\left(\lambda \right)}∕G_{\left(\lambda \right)}\right)$$

(16) $$M=\sqrt{s_{\left(\lambda \right)}^{2}+G_{\left(\lambda \right)}^{2}}$$

The phasor approach allows for easy deconvolution of the multiple fluorescence species and the linear combination converts the spectral phasor to quantitate information on the species. However, where the spectral phasor method shows its highest performance is when complex photophysics are involved, such as in solvatochromic probes, as we will discuss later [41,44,45]. 

## 3. Component Analysis

### 3.1. Two-Component Phasor Analysis

If the fluorescence decay originating from an image pixel consists of decays of two individual species (Figure 2a) and the phasor positions of those two individual contributing species are known, the image phasor points will lie along the line joining the phasor positions of those individual components (Figure 2b). The relative position of the image phasor coordinate can then be transformed to the fractional intensity of either species by calculating the distances from between the image phasor point to the component position [17,31] (Figure 2c). For a large image, the whole phasor cloud can be transformed by repeating these calculations. This approach is especially useful for autofluorescence metabolic imaging using NADH (reduced nicotinamide adenine dinucleotide) or FAD (flavin adenine dinucleotide). In the case of NADH, the fluorescence lifetime of free and protein-bound NADH are different, and they occupy different spots in the phasor plot. This fact is shown by the violet and red empty circles, respectively, in the example phasor plot (Figure 2b). The distribution of the phasor points originating from the sample can be calculated and because the whole phasor distribution can be transformed to a fractional intensity distribution of either free or bound NADH. Further statistical states can be employed to understand the significance of the metabolic changes as shown in Section 6.

### 3.2. Three-Component Analysis

In a pixel wherein three separate species are present (black dot, Figure 2d) the phasor of that pixel will be inside the triangle with vertices occupied by the phasor positions of the individual components, shown here by the red, green and blue circles (Figure 2d). The calculation of the three components is based on the projection of the phasor cloud to the line joining the two vertices (blue and green, Figure 2e,f) relative to the position of the third vertex (red) [32]. To do so, first a line connecting the red and black dots was extended to the line joining the vertices at the green and blue dots. The intercept point is used for the calculation of the contribution of the red component:

(17) $$Fraction_{Red}=\frac{Distance_{point\to intercept}}{Distance_{red\to intercept}}$$

To explain how the other two fractional intensities are calculated, let us assume that initially there were only two components (the green and the blue). The resulting phasor cloud will appear along the line joining them. Larger contributions of the red species will shift the phasor cloud towards the red point. The complete calculation for each vortex will result in three individual graphical representations, and three individual fractional intensity histograms.

The situation becomes simpler if two of the components are related. An example is free and protein-bound NADH (Figure 2e,f; blue and green circle, respectively). This distribution is shifted in the presence of a third species (red circle, Figure 2e,f). In terms of mole fractions (x), free and bound NADH are related by:

(18) $$x_{freeNADH}+x_{boundNADH}=1$$

This relationship simplifies the fractional intensities of free (or component C, blue circle, Figure 2e) and protein-bound NADH (or component B, green circle, Figure 2e):

(19) $$Fraction_{\mathrm{free}\;\mathrm{NADH}}=\frac{{\mathrm{Distance}}_{\mathrm{intercept}\to \mathrm{blue}}}{{\mathrm{Distance}}_{\mathrm{green}\to \mathrm{blue}}}$$

The fraction of a third component (red circle, Figure 2e,f) was obtained using a similar equation to that of Equation (17):

(20) $$Fraction_{LLS}=\frac{{\mathrm{Distance}}_{\mathrm{point}\to \mathrm{intercept}}}{{\mathrm{Distance}}_{\mathrm{phasor}\;\mathrm{position}\;\mathrm{of}\;\mathrm{LLS}\to \mathrm{intercept}}}$$

In terms of implementation, a graphical solution results in a much faster analysis. This analysis is carried out by choosing a cursor (orange cursors, Figure 2f) whose size relates to the statistical experimental error of each phasor point. Then, two types of scans are carried out. The line between blue → green (BC, Figure 2f) is then divided into multiple positions (orange solid cursors Figure 2f) and at each position, the phasor distribution is scanned along the line joining the red (point A, Figure 2f) and the orange points in the line BC (empty pink cursors and black lines). The number of phasor points are counted during the scan along the line joining point A to line BC and that number is plotted against the position of that particular orange cursor or the coordinate of line BC. This procedure yields the fractional intensities in the free/bound NADH plot.

### 3.3. Four-Component Measurement

Assuming that the phasor positions of individual molecular species are known and that there are indeed four components, the rule of linear combination of phasors requires calculation of the fractional intensity of each component at any given pixel. This rule assumes that the molecular species are not interacting. The four-component case results in a quadrilateral shape in the phasor plot (Figure 2g, phasor points 1, 2, 3 and 4). Here, there are four fractional intensities and to obtain more than four parameters, the phasor plot is transformed to higher harmonics. Each harmonic creates a new pair of equations and the system of four equations is solved by linear algebra methods [35].

The four equations for the linear combination of harmonics are written below [35]:

(21) $$f_{1}g_{1}^{h1}+f_{2}g_{2}^{h1}+f_{3}g_{3}^{h1}+f_{4}g_{4}^{h1}=G^{h1}$$

(22) $$f_{1}s_{1}^{h1}+f_{2}s_{2}^{h1}+f_{3}s_{3}^{h1}+f_{4}s_{4}^{h1}=S^{h1}$$

(23) $$f_{1}g_{1}^{h2}+f_{2}g_{2}^{h2}+f_{3}g_{3}^{h2}+f_{4}g_{4}^{h2}=G^{h2}$$

(24) $$f_{1}g_{1}^{h2}+f_{2}g_{2}^{h2}+f_{3}g_{3}^{h2}+f_{4}g_{4}^{h2}=S^{h2}$$

where *f_1_, f_2_, f_3_* and *f_4_* are the unknown fractional intensities, $g_{n}^{h1}$ and $s_{n}^{h1}$ are the *G* and *S* components of the first harmonic for the four phasor positions of the pure components and $g_{n}^{h2}$ and $s_{n}^{h2}$ are the *G* and *S* components of the second harmonic, respectively. These equations can be solved due to the orthogonality of the phasor harmonics.

The component analysis described above—especially two- and three-component analysis—was developed in the context of phasor FLIM; hence, it is described in this fashion. However, the same argument and idea is valid for the spectral phasor analysis, and can be expanded to understand and quantify the extent of fractional intensity contributions from multiple species differing in their fluorescence spectra.

### 3.4. Blind Component Analysis

The graphical decomposition explained above is only useful if we know the location in the phasor plot of the pure species. In the absence of that knowledge, and the normal ~100 photons/pixel collected in any given pixel, a pixel-wise “blind” method to resolve components and their fractional intensities is difficult using fitting methods for fluorescence lifetime decays. Using modern electronics where higher harmonics of phasor plots are readily available, this goal can be achieved. Below, we present a short description of the method and the mathematical approaches have been described in [38].

In the blind deconvolution approach, the phasor plots generated from multiple frequencies are combined to calculate the mono-exponential components [38]. This calculation is possible due to the mathematical constraint, wherein mono-exponentials will be at the universal semicircle which restricts the number of combinations that need to be computationally achieved. The main idea here is that provided that a phasor from a pixel is originating from multiple species, in every harmonic frequency of the phasor plots there are specific positions for that phasor point and its individual components. The linear combination of phasor space holds true for each of the harmonic frequencies and they can be solved either by using least squares methods for every possible combination of lifetimes, or by using minimization techniques such as the simplex algorithm or gradient based algorithms, or geometric approaches which are much faster and do not require initial guesses (for a two-component system) [38].

Mathematically, it relates to the following:

Pure mono-exponential decays lie on the universal semicircle (Equation (8)) and that can be used as a constraint on the conversion of the phasors in various harmonics. This allows for a simplified expression for where coordinates (g, s) from one harmonic (*n*) can be expressed in terms of another harmonic (*m*):

(25) $$g_{n}=\frac{m^{2}\cdot g_{m}}{n^{2}+\left(m^{2}-n^{2}\right)\cdot g_{m}}$$

This relationship between the different harmonics and Equations (11) and (12) (which can be described for multiple harmonics, as shown in Equations (21)–(24)) can be written for multiple harmonics and then a minimization algorithm can be used and extrapolated to obtain the fractional intensities and the phasor positions of unknown components.

#### 3.4.1. Two Components 

**Figure 3:**
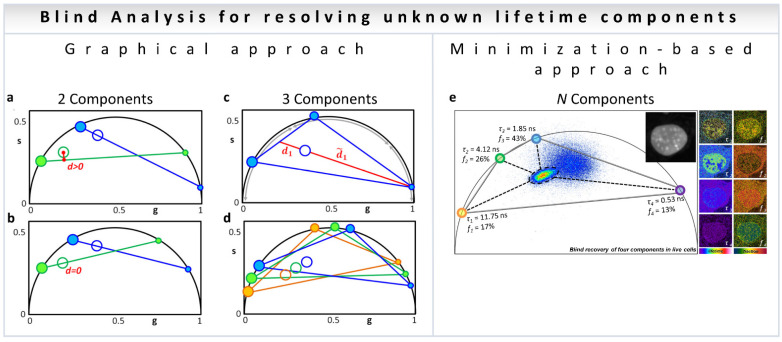
Blind component analysis for resolving unknown lifetimes in phasor plot—assuming the components have mono-exponential lifetimes. (**a**,**b**) Graphically finding two unknown lifetime components and their relative fractional intensities. (**a**) Universal circle is scanned for candidate components. For each position of the first component (small blue dot), a line is drawn through the data point (blue empty circle) and the second point is found where the blue line meets the universal circle (large blue filled circle). The second harmonic (*h*2) for the candidate components are obtained mathematically: small green dot and the large green filled circle. A line is drawn through them (green line). (**b**) Solution is the only lifetime pair that has the lines (blue and green) going through the data points for the two harmonics, that is, when the distance from the green line to the green empty circle is zero. The relative fractions are obtained by the ratios between the distances from the filled dots to the empty circles on both sides of the line. (**c**,**d**) Finding the three lifetime components in the universal circle. (**c**) Schematic configuration of three components generating a data point (ring in the middle). As an example, the ratio between the two distances is related to the intensity fraction (*f*) of the component located in the short lifetime region; *f*1 = *d*1/(*d*1 + *d*1). In the same manner, the other two fractions can be obtained for the other two components. (**d**) Solution to the three-component problem is the only combination of components such that the three fractions are the same in each of the triangles composed of the three harmonics (blue–green–orange). The short, medium, and long lifetime components in each harmonic are depicted by increasingly larger filled circles. (**e**) Minimization-based approach. This general algorithm can technically be solved the problem to *N* number of components depending on noise propagation in the multiple harmonics. (Panels **a**–**e** adapted with permission from [38]).

In the blind analysis of a two-component system [38], the graphical solution implements the following procedure. The open blue and open green in Figure 3a represents the experimental data at each pixel for the first (blue, *h*1) and second (green, *h*2) harmonic coordinates with two exponential components, filled blue (*h*1) and green (*h*2). A line is traced from a point on the universal circle (the small blue dot, Figure 3a) passing through the experimental data point (blue empty circle, Figure 3a) until it intersects the universal circle at a point (large filled blue dot, Figure 3a). The corresponding second harmonic positions of the two candidate lifetimes (green filled circles, Figure 3a) are calculated for each position of the candidate components (blue filled circles) while scanning the universal semicircle. The process is repeated for all possible positions of the candidates by scanning the phasor plots in two harmonic frequencies until the distance (*d*, Figure 3b) in the second harmonic is minimal. This presents the solution for blind deconvolution of two components (Figure 3b).

#### 3.4.2. Resolving N Components 

Similar to the two-component blind analysis, the algorithm involving three components involves scanning the three-dimensional space defined by the three components and finding the combination where the same fractional intensities satisfy the data in all three harmonics, with the same intensity fractions [38]. This idea is shown in Figure 3c,d, with empty circles for the experimental points and filled circles for individual components. The colors: blue, green and orange represent the harmonics *h*1, *h*2 and *h*3, respectively). 

An alternative to the graphical search is a minimization-based approach (Figure 3e). The minimization approach has the benefit of being applicable to any N number of components, although caution is required to avoid local minima [38]. Please see [38] for the details of the mathematical analysis.

Another important aspect of the analysis of N components and blind components is the propagation of noise in phasor space. Noises in lifetime components and harmonics are essentially different. In the case of noise in lifetime, added noise in one of the components has no effect on other components, due to the orthogonality of the phasor transformation, and does not propagate in other components. This is not true for noise added to the harmonics. The harmonics have both fractional intensity and lifetime components. Here, the calculated components are all affected by the added noise and these combinations of lifetime and fractional intensities are correlated. Please see Figure 5 and Tables 1–3 of the original reference [38] for further calculations. This propagation of noise in the harmonics essentially determines how many components can be calculated from the multiple component cases. Another aspect of these calculations is the extent of the difference between the lifetimes of the components and their phasor positions. If the lifetimes are well separated, then a better deconvolution can be achieved. An obvious case of inability of these deconvolutions of the phasor method is if one of the components has a lifetime that appears along the line joining the phasor position of two other components. In this case, the linear combination will assume the phasor position in the middle of this line to be related to the components whose phasor positions are at the end of the line. We have expanded the N component analysis to four components and work is underway to increase the numbers of components. However, the increasing noise and increasing spread of phasor in the higher harmonics will eventually make it difficult to increase the number of components.

### 3.5. Three-Component Analysis Based on Non-Euclidean Approach 

A different ideology based on non-Euclidean geometry using Mahalanobis distance [36,37] has been used to understand the effect of drugs on skin. Three components used here involved the free and protein-bound NADH and the drug itself. This approach involves defining a metric assigning the distance between an entire distribution and an observation phasor point. This procedure is based on the observation that free NADH and bound NADH can have a distribution of phasor points, as opposed to a single discrete location in the phasor plot. Mathematically, the Mahalanobis distance is defined as:

(26) $$D_{ij,\;ref}=\sqrt{{\left(\underset{x_{ij}}{\to }-\underset{\mu_{ref}}{\to }\right)}^{T}C_{ref}^{-1}\left(\underset{x_{ij}}{\to }-\underset{\mu_{ref}}{\to }\right)}$$

where, $x_{ij}={\left[G_{ij},S_{ij}\right]}^{T}$ is the observation from an image pixel (*ij*), and $\mu_{iref}={\left[G_{\mu },S_{\mu }\right]}^{T}$ is the mean of the reference set, and $C_{ref}$ is the covariance matrix.

The proportion of photons (*k_ij_*) arising from NADH in comparison from the third component, termed a sun filter (*SF*) in the original manuscript, is given by the ratio of the two Mahalanobis distances—sample to *SF* (*D_ij,SF_*) and the *SF* to *NADH* (*D_NADH,SF_*). The ratio is reversed and subtracted from 1 if it is below 0.5.

(27) $$k_{ij=}\frac{D_{ij,SF}}{D_{NADH,SF}}\;if\;k_{ij}\geq \frac{1}{2};\;\;and\;k_{ij=}1-\frac{D_{ij,NADH}}{D_{SF,NADH}}\;if\;k_{ij}<\frac{1}{2}$$

**Figure 4:**
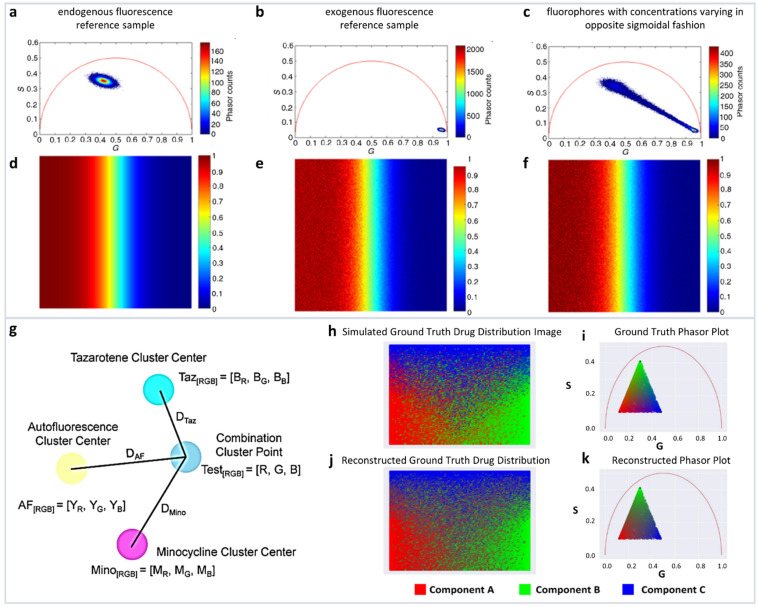
Multicomponent analysis based on non-Euclidean geometry. (**a**–**c**) Simulation to validate the proposed non-Euclidean separation algorithm. (**a**) Phasor plot of simulated endogenous fluorescence reference sample, (**b**) exogenous fluorescence reference sample, (**c**) fluorophores with concentrations varying in opposite sigmoidal fashion. (**d**–**f**) Three-component analysis of drug distribution using non-Euclidean geometry. Simulated endogenous fluorescence contribution to the test image. Estimation of endogenous fluorescence contribution computed using the traditional Euclidean method and using the proposed non-Euclidean method. (Panels **a**–**f** adapted with permission from [37]). (**g**–**k**) Distance-based method of assigning phasor plot pixel colors in a multicomponent fluorescence contribution analysis algorithm. (**g**) A sample setup of a distance calculation. Yellow, cyan, and magenta points → the center of exogenous (autofluorescence), and two endogenous reference clusters (TAZ and MNC), respectively, and blue point → a phasor point from the tissue sample. (**h**,**i**) Simulated ground truth tissue map of drug distribution and calculated phasor plot. (**j**,**k**) Reconstructed tissue drug distribution using the multicomponent phasor analysis algorithm and phasor plot. (Panels **g**–**k** adapted with permission from [36]).

It is clear that if the phasor is far from *SF* then the value of the number of photons is mostly related to *NADH*. This concept allows for pixel-to-pixel analysis of the various components. The simulations and recovery statistics using this method are shown in Figure 4a–f.

A modified method by the same authors used the linear combination to understand the distribution of minocycline (MNC), and tazarotene (TAZ), two drugs in skin samples. The authors used autofluorescence as one distribution and MNC, TAZ as the other two components. The mean G and S coordinates were calculated for each cluster. Then, variance of each cluster was determined by calculating the distance from each point in the reference cluster to the center point of that cluster, and then finally summing into the total variance. Finally, they modified the cluster center points by adding 25% of its fraction of total variance.

(28) $$y_{new}=y_{original}+\left(y_{original}\times 0.25\times variance_{cluster}\right)$$

The normalized contributions to individual phasor points from exogenous and endogenous clusters were then calculated by calculating three distances *D_Exo1_, D_Exo2_, D_Endo_* from a point P to the one endogenous and two exogenous cluster centers (Figure 4g). Following the linear additivity rules of phasor space, the individual contributions *C_Exo1_, C_Exo2_, C_Endo_* were defined as *1/D _Exo1_, 1/D _Exo2_, and 1/D _Endo_* (Figure 4g). This approach allows for the calculations of the individual contributions and the simulations are shown in Figure 4h–k.

## 4. Breakdown of Linearity and Improper Transformation to Phasor Plot

**Figure 5:**
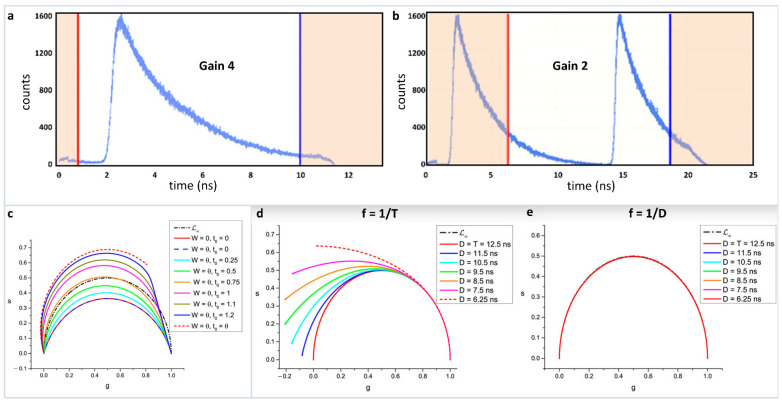
Improper transformation of fluorescence lifetime decays to phasor plots and their effects. (**a**) BH 830 card acquisition of the decay of a solution of Rhodamine 110 excited with a high rep laser at 80 MHz with two different settings of the card. (**b**) Gain of four gives a TAC time range of 12.5 ns. Due to the non-linearity of the TAC at early and later times, only one portion of the decay, indicated by the red and blue lines, is valid giving a total range of about 9 ns, which is insufficient to cover an entire period of the laser. (**c**) Setting the gain at two gives a total larger range (25 ns) but only one-part corresponding to 12.5 ns is used (indicated by the red and blue lines) which covers an entire period of the excitation laser. The orange shaded parts of the decay are not used. (**c**) Effect of a decay offset on the ℒN [W] in the simple case where the gate width W is equal to the gate step θ. The calculations were made with T = 12.5 ns, N = 10 (i.e., θ = 1.25). The standard ℒ∞ (semicircle) is represented as a black dotted-dashed curve. ℒN (plain red curve) and ℒN [θ] (plain dark blue curve) are identical, yielding a red/dark blue dashed circular arc. As the decay offset t0 increases (with t0 < θ), ℒN [W] progressively rotates towards and is deformed into ℒN [θ], which is a circular arc (red dashed curve). (**d**) Continuous phasor of truncated decays. T = 12.5 ns and the calculated loci of continuous phasors of ungated PSED (SEPL) are a function of the observation duration D ≤ T when f = 1/T (**d**) or f = 1/D (**e**). In the latter case, all curves are identical to ℒ∞. However, if the phasor frequency is chosen to be the fundamental Fourier frequency f = 1/T, the SEPL increasingly departs from the ℒ∞ as the observation duration D decreases. Please see Figures 7 and 8 from the original [39] for more details and explanation of the mathematical quantities. (Panels **a**,**b** and **c**–**e** are adapted with permission from [40] and [39], respectively).

As explained in Equations 1–4, phasor positions can be calculated from both time and frequency domain measurements. In the frequency domain, the transfer to the phasor plot involves only knowledge of the phase shift (ϕ) and the relative modulation (M). In that sense, it is a very straightforward transformation. However, in the time domain attention must be paid for the proper transformation procedure. Phasor transformations requires a repeating signal and full decay information. For an intensity decay, this process involves having the complete shape of the decay, i.e., the intensity decaying to baseline. In solution measurements where the laser frequency is often decreased, commonly using a pulse picker, this information is not difficult to obtain. In measurements on biological samples, the laser frequency is often set at 80 MHz for a Titanium-Sapphire laser and only the fundamental laser frequency is used. This frequency results in a gap of 12.5 ns between consecutive pulses. Due to the non-linearity of the time to amplitude converter (TAC), often only 9–10 ns of this time can be used. The intensity decays of biological fluorophores with lifetime above 2 ns do not reach background levels during this timescale (here exemplified by using Rhodamine 110 in water with a 4.0 ns lifetime (Figure 5a, [40]). The transformation of this decay to phasor can lead to improper transformation with a resultant breakdown of phasor linearity. Another way to circumvent this problem can be using a lower TAC gain wherein the observation window is 25 ns (Figure 5b) and in this case a window can be selected in which part of the decay from the first pulse and part of the decay from the second pulse is present. This procedure results in a repeating signal with complete decay information where proper phasor transformation can be carried out.

Further complications can arise when transformations happen from time gated measurements [39]. The recent work by Michalet et al., have shown caution is required for the proper phasor transformation and for incomplete decays. This work combines different scenarios related to time gating—continuous decays, continuous vs. discrete and ungated vs. gated phasors. Transformation of both continuous and discrete decays were looked at under different scenarios: (i) periodic single-exponential decays (PSEDs), (ii) PSEDs with single-exponential IRF, (iii) square-gated PSEDs, and (iv) square-gated PSED with single-exponential IRFs. The author has also looked at the effect of truncated data and the offset in rise time under these scenarios. For example, the effect of a decay offset on discrete phasor calculations can be seen in Figure 5c. It can be seen how the offset modifies the phasor trajectory of the single-exponential decays. Similarly, Figure 5d shows the effect of truncation for continuous phasor calculation. As the truncation increases, the phasor plots deviate from the expected phasor plot, where the period is set as T = 12.5 ns and the truncated part of the decay D ≤ T. If the observable duration D = T, then the single exponential decays follow the universal semicircle (Figure 5e).

The proper transformation of the intensity decays for FLIM results is the linear additivity of phasor space, and as mentioned above that linearity can be lost on improper conversion. Similarly, if there are extra constraints on the phasor transformation, then the linearity will also be lost. An example is the diffusion phasor [46], which by transforming fluorescence autocorrelation decays measured from a very short intensity time trace at each pixel of an image converts them to phasor space. Here, an additional constraint ($g^{2}+s^{2}=0.5$) is added so the calculation and the linearity of the phasor space is not valid. Improper transformation of spectral phasor can be found when part of the spectral relaxation is cut off. In this case, the linear properties of the phasor space are eliminated from the phasor space. For the transformation to be valid, full spectra need to be transformed; the use of partial spectra, where the center position in wavelength and spectral width are not correct, can lead to loss of linearity.

## 5. Reciprocity Principle and Multidimensional Phasor Approach

**Figure 6:**
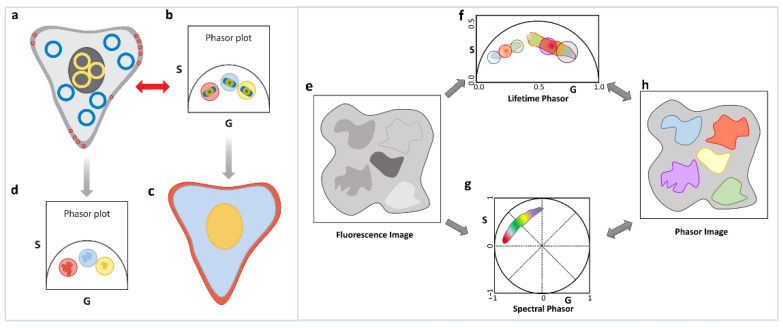
Reciprocity principle and multidimensional phasor approach. (**a**–**d**) Reciprocity principle. Phasor signatures from different parts of the image (**a**) can be segregated (**b**). Selection of these different phasor clouds (red, blue and yellow circle, (**b**) allow the image points they originated from to be selected and the image to be colored accordingly (**c**). Similarly, parts of the image can be selected (blue, yellow and red cursors, (**a**) and the corresponding phasor points can be highlighted (**d**). (**e**–**h**) Multidimensional phasor approach. When the exact same area of a sample is imaged (**e**) in different moieties using phasor approach, phasor FLIM (**f**) and spectral phasor (**g**), then the phasor image (**h**) can be color-coded according to one selection, in this case, lifetime phasor (**f**). Using the reciprocity principle, the spectral signatures of those lifetime phasor selections can be identified (**g**).

A final observation and very important principle that we want to highlight is the reciprocity principle [1] and the multidimensional phasor plots [41]. Although strictly not related to the linear additivity of phasor, we feel that this principle is the basis of the increasing use of the phasor approach. If the decays from various areas of an image have different decay characteristics, then the resulting phasor points will appear in segregated areas of the phasor plot (Figure 6a–d). The segregation in the phasor plot is due to different parts of the image having different lifetimes and can have a filtering effect that allows areas in the image with similar decay characteristics to be identified by selecting the segregated parts of the phasor plot (Figure 6a–d). Alternatively, the phasor plot has embedded information about the location of the image pixels giving rise to each phasor point. This correlation between image pixels and their specific phasor positions results in the reciprocity principle (Figure 6a–d). Using this principle, a part of the phasor cloud can be selected, and the corresponding image pixels can be highlighted. Based on the reciprocity principle parts of the image can also be selected and the corresponding phasor points can be highlighted. These operations allow for easy and fast interrogation of both the phasor and image space and greatly increase the efficacy of the analysis.

The multidimensional phasor approach is based on this reciprocity principle. If multiple phasor properties can be calculated for the same image (Figure 6e), e.g., lifetime and spectral phasor (Figure 6f,g), then the reciprocity principle allows for selection of a set of phasor points in one dimension (let us assume lifetime, master phasor). This results in the image pixels those phasors originated from being highlighted (Figure 6h), and finally the properties of those image pixels in another dimension are shown (in this case spectral [26,41], see Section 6.2). Using this approach, we can combine excitation-emission spectra, many lifetime channels, as well other dimensions such as diffusion phasor or anisotropy/polarization. Thus, the reciprocity principle can enable us to ask questions about multiple different properties of the various fluorescent species and can help us to possibly identify various species by combination of their various spectroscopic characteristics. The possible applications, including autofluorescent species in biological systems, can possibly let us fingerprint various species.

## 6. Results from Biological Measurements

### 6.1. Results of Multicomponent Analysis in Phasor-FLIM Imaging

The phasor-FLIM approach has been used in autofluorescence metabolic imaging, utilizing the inherent fluorescence of natural biomarkers of cellular metabolism: NADH and FAD. NADH and FAD are enzymes cofactors involved in redox metabolic reactions. These endogenously fluorescent cofactors allow noninvasive metabolic imaging of cells and tissue without perturbation of the samples. NAD is not fluorescent, so NADH is the contributor to the autofluorescence. For FAD, the situation is the opposite and FADH_2_ is the species that is not fluorescent. NADH and FAD differ in their excitation and emission spectrum, and each of them exists in two spectroscopically distinctive states: free or bound to proteins, and these two states differ largely in their fluorescence lifetimes [22,34] and quantum yields [47]. The lifetime of free NADH is shorter (0.4 ns) than the protein-bound NADH (1.3–9 ns) [22,34]. Contrary to this case, free FAD has a longer lifetime (3–4 ns) compared to its protein-bound state (<1 ns) [48].

**Figure 7:**
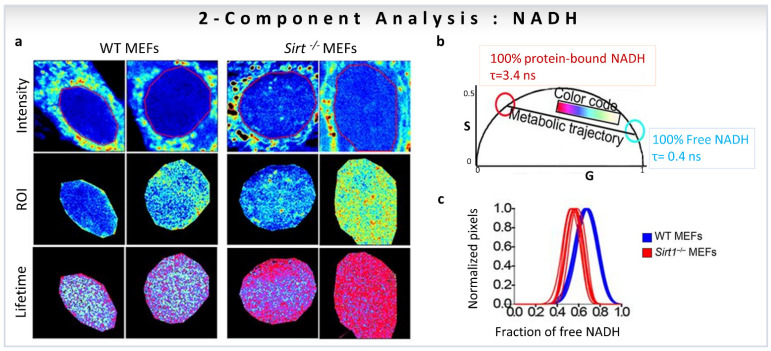
Biological examples of fractional intensity contributions of two species based on linear combination of phasor-FLIM. Two-component analysis (known lifetimes of the components) using a graphical approach for metabolic imaging using free to bound NADH. (**a**–**c**) Nuclear NADH metabolism is dependent on the activity of Sirt1. (**a**) Intensity and lifetime (FLIM) images for WT and *Sirt*^−/−^ MEFs. Two representative images per condition are shown. (**b**) Phasor plot shows the color scale used to visualize the fraction of free/bound NADH along the metabolic trajectory in FLIM images. (**c**) Histograms show comparative analyses by overlapping the fractional free NADH distributions from WT (cyan) and *Sirt*^−/−^ (red) MEFs. Free NADH fraction is represented in the x axis, where 1 = 100% free NADH. (Panels adapted with permission from [17]).

The ratio of free-to-protein-bound NADH has been associated with the NAD+ to NADH redox ratio, and the amount of free and protein-bound NADH can be correlated with changes in the energy metabolism of cells [49,50,51,52]. Hence, it is important to distinguish and calculate the fraction corresponding to each of the NADH states. The linear addition property of the phasor space with FLIM images has been extensively utilized to calculate the shift between free and protein-bound NADH in cells and tissues to correlate with changes in cellular metabolism [5,6,19,20,32,53,54]. The line joining the phasor positions of free and protein-bound NADH is called metabolic trajectory [19] (black line, middle panel, Figure 7b) and a shift of the phasor cloud along this trajectory is indicative of more oxidative phosphorylation (more towards bound NADH, red circle, Figure 7b) or more glycolysis (towards more free NADH, cyan cursor, Figure 7b) [6,22].

One of the first studies to quantify the fractions of individual components based on linear additive properties of phasor-FLIM focused on the role of the NAD-dependent deacetylase SIRT1 in the subnuclear distribution of free and protein-bound NADH [17]. FLIM images of WT vs. Sirt1^−/−^ MEFs in the cell nuclei were taken with an NADH filter (460/80 nm) to collect the contribution from free NADH (0.4 ns, cyan/white circle) and protein-bound NADH (3.4 ns, red circle), and avoid FAD fluorescence (Figure 7a,b). Figure 7b shows the positions of NADH species on the universal circle in the phasor plot, and the color-code used to visualize the metabolic trajectories. The use of the two-component graphical analysis (for calculations, see Section 3.1) of the FLIM data allowed a comparison between the relative fractional intensities of free and protein-bound NADH in WT and Sirt1^−/−^-MEFs (Figure 7c). The fraction of protein-bound NADH is significantly higher in Sirt1^−/−^MEFs compared to WT MEFs (Figure 7c). Additionally, NADH subspecies are distributed in distinct nuclear territories with protein-bound NADH located at the nuclear periphery, whereas free NADH concentrates in the nuclear interior (Figure 7a). The absence of SIRT1 reorganizes NADH metabolism in the nucleus (Figure 7a). The conclusion is that NADH has a specific distribution in the nucleus, and that nuclear NADH metabolism is associated with SIRT1 function.

**Figure 8:**
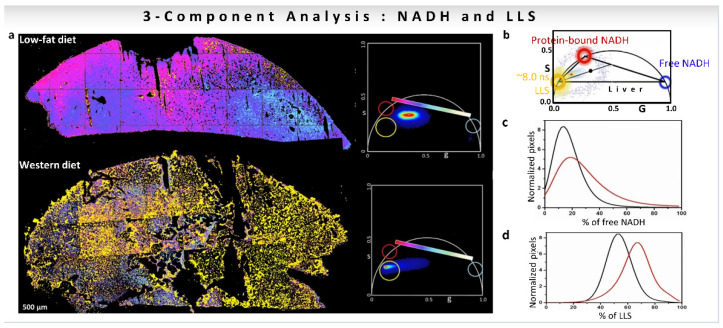
Three-component analysis (known lifetimes of the components) using a graphical approach for understanding of effect of diet on mice liver. (**a**–**d**) Quantification of the presence of oxidized lipids in mice fed with Western diet vs. low-fat diet using three-component analysis. (**a**) Color-coded phasor-FLIM liver images of mice fed with low-fat diet (LF) or Western diet (WD). The free-to-protein-bound ratio (not including LLS) is color-coded using a magenta to cyan colormap with a yellow overlay for LLS. The corresponding phasor plots are shown for each condition. (**b**) Blue, red and yellow circles on the phasor plot represent the phasor positions of free and protein-bound NADH, and long lifetime species (LLS), respectively. Histograms showing the percentage of free NADH (**c**) or LLS species (**d**) for the LF (black) and WD (red) diet, respectively. (Panels adapted with permission from [32]).

The calculation of changes along the metabolic trajectory becomes more difficult if other fluorescent species appear in the NADH detection channel, shifting the phasor distribution. This situation is the case with the long lifetime species (LLS, ~8 ns) that are present in cells and tissues, which have been associated with oxidized lipids (Figure 8a,b [19,32,33,55]). The presence of LLS species shifts the phasor position of the pixels with different free/bound NADH ratios toward the LLS location in the phasor plot (Figure 8b, [32]). As Figure 8b shows, for a three-component system, the phasor points will be inside the triangle formed by the original components. The decomposition of the relative contribution of each species at each pixel of the image can be carried out using a three-component phasor analysis (for calculations see Section 3.2). This simple graphical construction using the phasor representation offers a great advantage in solving the problem compared to using the decomposition of the decay in exponentials. The calculation of a metabolic index in the presence of LLS was performed on mice livers fed with low-fat (LF) or Western diet (WD, high fat content) as shown in Figure 8a. FLIM images were taken with a restrictive emission filter to have only the contribution from free NADH (~0.4 ns, blue circle), protein-bound NADH (3.4 ns, red circle), and the long lifetime species (~8 ns, yellow circle). The positions in the phasor plot of these species are shown in Figure 8b. The corresponding phasor plots for LF and WD samples are shown in Figure 8a (right). The fractional intensities obtained when applying a three-component analysis show a larger fraction of free NADH in WD mice samples compared to LF samples (Figure 8c). Since the distribution is shifted along the metabolic trajectory towards an increase in free NADH, a high-fat diet in mice introduced a more reducing condition, and more glycolytic metabolism. Additionally, the fraction of LLS is higher in WD than LF mice (Figure 8d) [33].

**Figure 9:**
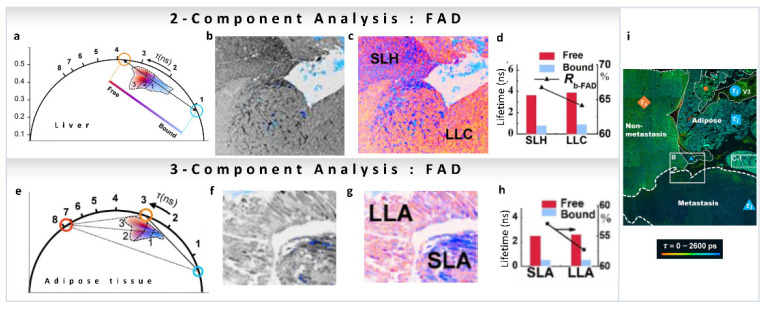
(**a**–**e**). Metabolic imaging of FAD using multiple components. FAD FLIM data of hepatocytes and cancer cells of metastatic pancreatic cancer (panels adapted with permission from [48]). (**a**) Phasor plot showing linear combination of two components, short lifetime (protein-bound FAD, blue cursor) and long-lifetime (free FAD, orange cursor). (**b**) Grayscale (accumulated photon counts) of hepatic cells. (**c**) Pseudo-color image showing different lifetime species in the short-lifetime hepatocytes (SLH) and long-lifetime cancer cells (LLC). (**d**) Lifetimes and ratios (*Rb-FAD*) of the components. (**e**,**f**) FAD FLIM data of adipocytes of metastatic pancreatic cancer. (**e**) Phasor plot showing three-component analysis, short lifetime (protein-bound FAD, blue cursor), long-lifetime (free FAD, orange cursor) and another much longer lifetime component (red cursor). (**f**) Grayscale (accumulated photon counts) of adipose cells. (**g**) Pseudo-color image showing different lifetime species in the short-lifetime adipose (SLA) and long-lifetime adipose cells (LLA). (**h**) Lifetimes and ratios (*Rb-FAD*) of the components. (**i**) Metabolic features of liver section (large-field image) of metastatic colonization revealed using FLIM. (Panels adapted with permission from [48]).

As we have mentioned, many reports have been published on the variations in metabolic pathways revealed by NADH FLIM, but this is not the case for FAD. Recently, FLIM FAD autofluorescence combined with the phasor approach has been used by Shen et al., to evaluate pathological features of metastatic colonization of pancreatic cancer [48]. FAD shows a reverse tendency compared to NADH, has a long lifetime in the free state (3–4 ns) and a shorter lifetime in the protein-bound state (<1 ns, Figure 9a). FLIM intensity images of hepatocytes and cancer cells of metastatic pancreatic cancer are shown in Figure 9b (intensity image), and the phasor color-coded image in Figure 9c. Images were taken with an excitation wavelength of 450 nm and an emission filter of 550/40 nm to collect the fluorescence from free FAD (~4 ns, orange circle) and protein-bound FAD (<1 ns, cyan circle) avoiding NADH fluorescence (Figure 9a,b). Again, a two-component analysis (for calculations, see Section 3.1) of the phasor plot was performed to obtain the FAD free-to-protein-bound ratio. A shift towards free FAD along the metabolic trajectory was observed, indicating a change from oxidative phosphorylation to glycolysis, which the authors suggest is related to cancerous behavior of the cells (Figure 9d). In adipose tissue of metastatic pancreatic cancer, long-lifetime species (~7.8 ns) were also observed in the FAD channel (Figure 9e–h). In this case, a three-component decomposition (for calculations see Section 3.2) was performed, and an increase in the fraction of free FAD was observed (Figure 9h). Figure 9i shows whole-area analysis of free/bound state of intracellular FAD metabolism in the tumor microenvironment, where cancer-associated optical signatures were associated with FLIM. These metabolic features demonstrate that changes in metastatic colonization can be probed by the characteristic parameters of free and protein-bound FAD.

**Figure 10:**
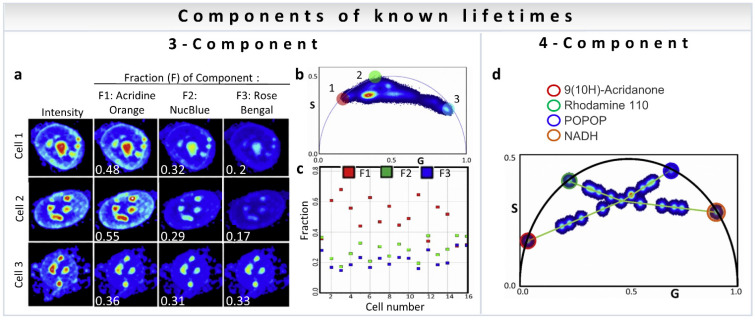
Multiple-component fraction analysis (known lifetimes of the components) to calculate average from an image. (**a**–**c**) Three-Component fraction analysis (known lifetimes of the components). Obtaining intensity fractions of three nuclear dyes mixed in live cells with three-component analysis. (**a**) Representative images of three different cells, all of them stained with the three nuclear dyes: Acridine Orange (Fraction 1, F1), NucBlue (F2) and Rose Bengal (F3). Total intensity (column 1) and normalized recovered images of the intensity fractions (average fraction written in white) of the three dyes are shown in columns 2–4. (**b**) Phasor plot corresponding to 47 different cells. The colored circles indicate in the phasor plot the phasor position for the first harmonic at 80MHz of Acridine Orange (red circle), NucBlue (green circle) and Rose Bengal (blue circle). (**c**) Fractional intensity values obtained with the three-component analysis of 16 cells. (**d**) Four-Component analysis (known lifetimes of the components) using harmonics. (Panel adapted with permission from [35]). Phasor plot corresponding to 22 different mixtures of 9(10H)-Acridanone (red circle, 12 ns), Rhodamine 110 (green circle, 3.8 ns), POPOP (blue circle, 1.4 ns), and NADH (orange circle, 0.4 ns) (phasor positions of the pure single exponential species on the universal semicircle are shown by circles). To solve the four-component system, the phasor plot was measured at 80MHz (first harmonic) and 160 MHz (second harmonic, not shown). (Panels adapted with permission from [35]).

A point to note is that the analysis methods discussed above are not restricted to metabolism measurements. The three-component analysis can be used to calculate the fractional intensity contribution of nuclear dyes mixed in live cells [35]. Figure 10a shows representative intensity images of three cells with the addition of three dyes (Acridine Orange, F1; NucBlue, F2; and Rose Bengal, F3). The fractional intensity of each component was calculated and is shown in white. The phasor plot of all cells together is shown in Figure 10b, with the phasor positions of component 1: Acridine Orange (red circle), component 2: NucBlue (green circle), and component 3: Rose Bengal (light blue circle). The results of fractional intensity calculations using a three-component analysis of 16 cells are shown in Figure 10c.

**Figure 11:**
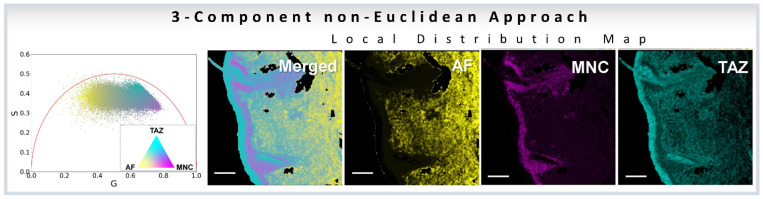
Quantification and visualization of minocycline (MNC), tazarotene (TAZ) in ex vivo skin samples. Quantification of the drug distribution for the three components: MNC, TAZ and autofluorescence based on the ono-Euclidean geometry results in distribution patterns for the three components, as shown by yellow for AF, cyan for TAZ, and magenta for MNC, respectively. The scale bar is 100 μm. (Figure adapted from [36]).

Similarly, the three-component analysis using non-Euclidean approach (Section 3.5) has been used to understand the effect of dyes in skin. In the first paper the authors used a three-component analysis to understand the effect of chemical sun filters, i.e., active ingredients of sunscreen lotions, to understand the topical distribution of the sun filters in ex vivo skin samples [37]. In the second work the same authors used the three-component analysis for understanding penetration and distribution of a topical gel (BPX-05), that can be used to treat moderate to severe acne vulgaris by delivering the active ingredients: topical antibiotic minocycline and the retinoid tazarotene directly to the pilosebaceous unit of the dermis. Here, minocycline (MNC), tazarotene (TAZ), and autofluorescence from NADH were treated as the three components [36] (Figure 11).

Resolution of the fractional intensities of four components in one pixel is possible in FLIM images using the phasor approach with a simple algebraic algorithm developed by Vallmitjana et al. [35]. This method is based on the law of linear combination of components that is valid after transformation of the decay curves to phasor space for each pixel in the image. For four-component decomposition, the phasor position of all individual molecular species has to be known for both first and second harmonics, available using modern electronics (for calculations see Section 3.3). Figure 10d shows measurements of mixture of dyes of different chemical compositions and lifetimes: 9(10H)-Acridanone (12 ns), Rhodamine 110 (3.8 ns), POPOP (1.4 ns), and NADH (0.4 ns), where the mixture at the center of the four green lines was obtained by adding four components of equal fractional intensity. Using linear algebra, it is possible to resolve four components in one pixel given the position of the phasors of four independent components in mixtures of dyes.

**Figure 12:**
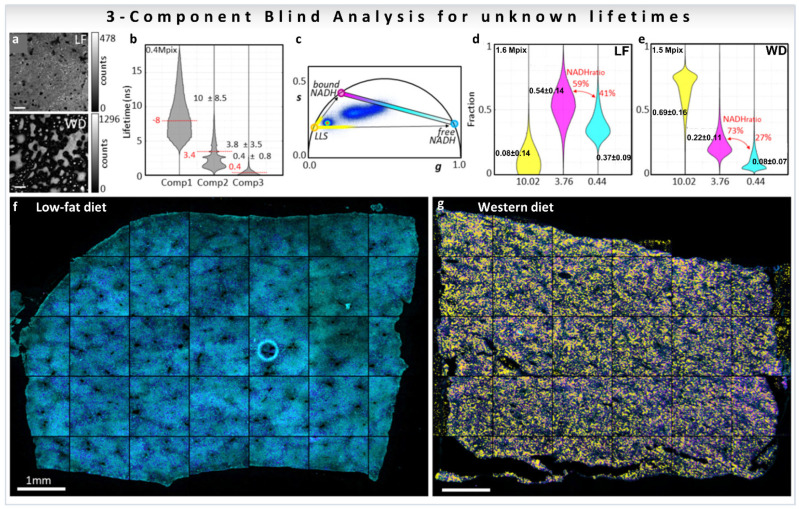
Biological example of three-component blind analysis (unknown lifetimes of the components) using harmonics of phasor. Study of metabolic changes in liver tissue samples using three-component blind recovery analysis. (**a**) Intensity images of livers of mice fed with low-fat diet (LF, top) or Western diet (WD, bottom) (**b**) Recovered lifetimes using three blind components are represented as gray violin plots and the median ± SD. Expected lifetime values for individual species are shown with a horizontal red line. (**c**) Phasor plot for the first harmonic (second and third harmonics phasor plots are not shown). Colored circles on the universal circle indicate the recovered first harmonic phasor position of free NADH (cyan), protein-bound NADH (purple), and LLS (yellow). Fractional intensity distributions of the three components in LF (**d**) and WD (**e**) samples. Color-coded phasor-FLIM liver images of mice fed with LF (**f**) or WD (**g**). The free-to-protein-bound ratio (not including LLS) is color-coded using a magenta to cyan colormap with a yellow overlay for LLS (with values above 75%). (Panels adapted with permission from [38]).

The biological problem of determining the free-to-protein-bound NADH ratio in the presence of LLS was addressed using a blind three-component decomposition [38]. The method uses the phasor approach and measurements of the decay at phasor harmonics 2 and 3. The blind component algorithm was performed in mice liver samples fed with low-fat (LF) or Western diet (WD) that causes lipid droplet accumulation in liver (Figure 12a, for calculations see Section 3.4.2), and the three median recovered lifetimes were [38] 10.0, 3.8, and 0.4 ns (Figure 12b). The lifetimes are displayed as colored circles on the universal circle on the phasor plot in Figure 12c. The three distributions are close to the expected lifetime values 7.8, 3.4, and 0.4 ns, previously shown in this review in Figure 7b and Figure 8b, proving that the blind component analysis can calculate correct lifetimes for the emitting species without knowledge of the system. Afterwards, the three recovered lifetimes were used to calculate the fractional intensity contribution of each of the components in every pixel of the image (LF; Figure 12d, WD; Figure 12e), finding that LLS are 8.6 times more abundant in the WD liver than in the LF as previously reported (Figure 8d, [32,33]). The three recovered fractions were finally used to obtain ratios of free/bound NADH excluding the presence of the LLS (medians of the value shown in red Figure 12d,e). The free NADH ratios obtained were 41% for LF and 27% for WD, meaning that there is more protein-bound NADH in the WD liver than in the LF. Images in Figure 12f,g are color-coded using a colormap (magenta to cyan on the phasor plot, Figure 12c) along the metabolic trajectory, for the ratio between the free and bound NADH lifetimes, on which the presence of the LLS were overlaid with a semitransparent yellow layer for the LLS fraction over 75%. This blind analysis can then be used to obtain quantitative information from phasor-FLIM images, including quantification of LLS, which are a signature of lipid oxidation that is present in fat droplets. Moreover, this new analysis allows for quantification of changes in metabolism even in samples where the third species (LLS here) modifies the phasor plot.

### 6.2. Spectral Phasor: Uses, Considerations and Applications

**Figure 13:**
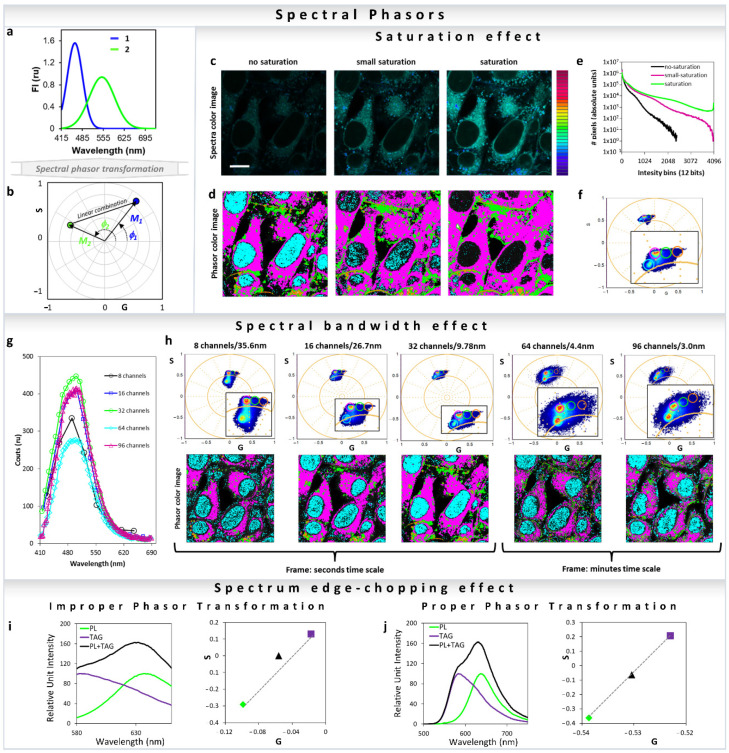
Spectral phasor transformation properties and important considerations. (**a**,**b**) Simulation of two gaussian curves with different centers of mass and FWHM (**a**), and the transformation into the spectral phasor plot (**b**). Note that while the blue curve has a longer M and short Φ (meaning bluer and narrow spectra), the green curve has a shorter M and larger Φ (meaning greener and broader spectra). The line that joins the two components represents the linear combination for all the different fractions between component 1 and 2. (**c**–**f**) Effect of the spectral saturation on the spectral phasor transformation. (**c**) Spectral and (**d**) pseudo-color images of HeLa cells stained with ACDAN (5 µM final concentration), imaged at different laser powers (with increasing saturation from left to right). Scale bar 10 μm. (**e**) The corresponding intensity histograms. (**f**) Based on a set of cursors in the phasor plot for the no-saturation condition, the effect of saturation and changes in the location of the phasor cloud by aberrant transformation can be seen. The phasor plot consists of the clouds from the three images (no-saturation, small-saturation, saturation). (**g**,**h**) Effect of the spectral bandwidth on the spectral phasor transformation. (**g**) The average spectra of the pixels from each image are shown here (8, 16, 32, 64 and 96 channels, respectively). Note that counts were kept in a similar range for comparison purposes. The data were acquired using a 32-channel spectral on the Zeiss LSM 710, therefore by default it records spectra using 32 parallel channels. Note that increasing resolution (increasing number of channels and decreasing the bandwidth per channel) of the phasor transformation results in a broader cloud (due to the poorer S/N per channel). The second noticeable difference is the time it takes to acquire the image due to the wavelength scanning needed for higher resolutions. Analyzing the image quality for the different configurations, it is simple to note that 32 channels is optimal in terms of image speed and resolution. (**i**,**j**) Improper spectral phasor transformation by the spectrum edge-chopping. In this example fluorescence spectra from Nile Red in phospholipid (PL) or triglyceride (TAG) (obtained from https://www.thermofisher.com/order/fluorescence-spectraviewer#!/, accessed on 25 November 2021) were transformed into spectral phasors using Equations (13) and (14). (**i**) the spectral range was chopped from both sides. The linear combination breaks down in this case (black triangle, 50:50 of each) due to the improper transformation. (**j**) Same set of data, but using the entire spectra results in a proper transformation and the linear combination is valid.

All major fluorescence microscopy vendors offer different arrangements for hyperspectral detection. Companies such as Nikon and Zeiss use PMT-arrays with different number of actuators (16 to 32 channel), which results in parallel collection of all spectral colors during the same dwell time and with a single illumination process. Other companies use single or two detectors to collect the emission spectra in steps with variable windows (Leica and Olympus). In this last example, the collection of the spectra takes longer because each step of the image is recorded individually, resulting in *n* numbers of decided illumination steps, where the resolution of each step can be defined by the users. One last option is the use of a spectrograph and a camera, when emission light is projected as a line in a camera chip and the resolution reaches 1 nanometer. In all cases, the light is split by a dispersive element such as prism or grating and then projected to the PMT-array, camera or to a PMT. Traditionally, the analysis of the spectral data requires methods such as component deconvolution or principal component analysis. In all of these analysis methods, knowledge about the system in study is required, and regardless of the methods used, either a physical or a mathematical model is assumed. In the spectral phasor space, no assumption is needed and analysis (Figure 13a) is model-free, similar to the phasor for the time-resolved fluorescence. Any spectra should have a position in the four-quadrant phasor plot, which is related to the center of mass and FWHM [1] (Figure 13b) (Equations (13) and (14)). It is simple to obtain the fractional intensities of each component when the positions of the single components are known, using the method described in Section 3 for the lifetime [32,35]. However, the most important applications are in situations where it is not trivial to obtain the “pure” component, and therefore use of regular deconvolution is impossible and/or inappropriate. For instance, with solvatochromic probes such as LAURDAN and Nile Red, there is no possibility to reproduce the exact environment present in cell membranes or lipidic organelles [26,43,44,45,56]. Before highlighting this application, let us cover some important considerations needed for a proper spectral phasor transformation. One very important and easy to understand consideration for improper transformation is to avoid saturation in spectral acquisition. Considering all new instruments that enable 16 bits resolution (65536 intensity values) it is very hard to saturate a pixel, but still it is recommended to acquire images with an active histogram or high–low function to avoid making this irreparable data mistake. In Figure 13c, one can see the effect on the phasor cloud distribution and position due to different levels of saturation. Increasing saturation can cause abhorrent phasor transformation loss of sensitivity (Figure 13c–f). The effects of the spectral bandwidth on the spectral phasor transformation are shown in Figure 13g,h. The phasor mapped images and the graph (Figure 13g) show that in Zeiss LSM 710 32 channel spectral detection gives the best result. This is due to the fact that there is an optimum number of channels which gives the best phasor transformation. Increasing numbers beyond that decreases signal to noise (S/N) in each channel, to an extent that the phasor cloud becomes broader.

Similar to the improper phasor transformation of FLIM data shown in Figure 5, improper transformation of fluorescence spectral data to the phasor space can also create a breakdown of the linear combination properties (Figure 13i,j). Transformation of truncated spectra (Figure 13i) results in breakdown of the linearity; however, by completely converting the full spectrum maintains the linear additivity (Figure 13j).

**Figure 14:**
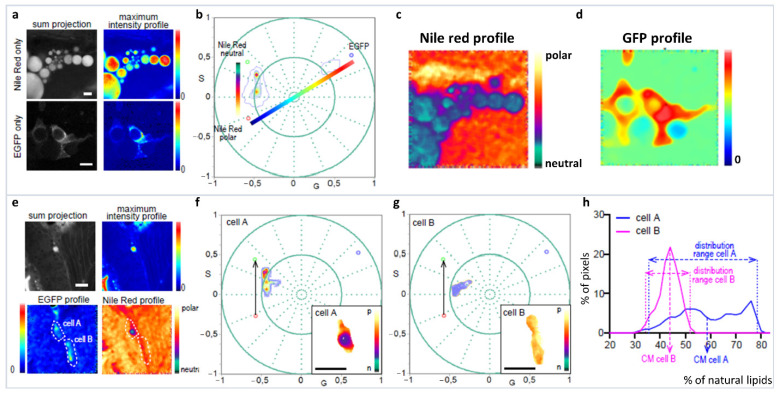
Analysis of the lipid metabolic profile of EGFP+ cells in the zebrafish line with the transgene fabp4 (−2.7): EGFPcaax. (**a**–**d**) Schematic representation of the three-component analysis for the Nile Red fluorescence in the presence or absence of the EGFP fluorescence. EGFP− cells let us define the neutral-polar Nile Red trajectory and EGFP+ cells without Nile Red, allowing us to identify the phasor position of the third component (GFP, identifying the specific cell-adipocyte). Notice that after three cursors were defined, a triangle has all possible linear combinations, and the third component was used to analyze the Nile Red information from the cells of interest. Two scales of colors were defined for the Nile Red profile and the EGFP profile. (**e**–**g**) Examples for the analysis of the Nile Red profile in two cells from the zebrafish larvae with the transgene fabp4(−2.7): EGFPcaax at 8 dpf. Notice that the Nile Red spectrum characteristic in the EGFP+ cell is pulled towards the third component, which enables the identification and analysis of the Nile Red profile for these cells avoiding confusing the information coming from Nile Red in GFP− tissue. (**h**) “Nile Red axis” is defined as a normalized fraction of pixels along the Nile Red profile. The histogram shows the percentage of polar lipids in the region of the cell analyzed. Scale bars: A: 20 μm; B: 50 μm. (Panels adapted with permission from [56]).

The spectral phasor approach to hyperspectral imaging analysis is highlighted using a complex problem, where Nile Red (NR) polarity profiles from different cellular environments are required to be analyzed from a specific cell line (adipocyte) on an in vivo zebrafish [56]. First problem to be faced is the analysis of Nile Red in the different subcellular organelles and membranes. Here, deconvolution is not recommended as the component maximum and FWHM are not available. In comparison, ratiometric analysis or generalized polarization (GP) [57] assumes a two-state system without the possibility of including a third component in the model (such as autofluorescence). To identify our cells of interest, they are labeled with green fluorescent protein positive (GFP+), and this results in the requirement of having a third component (GFP) in the spectral phasor analysis. The spectral phasor simplifies this complicated system without the need of detailed knowledge about Nile Red (NR) spectroscopy, and separating out the interference of GFP. First the NR dimension in tissue without a third component (GFP−) is defined, showing a discrete environment in the bluer side of NR emission, related to lipid droplet (apolar environment) (Figure 14a–d). Then, there is a trajectory from the end of the apolar environment towards the redder part of the spectra, which is associated with cellular membranes (liquid-order/disorder, polar environment) (Figure 14e–g). The third component (GFP) identifies our cell of interest (GFP+ cells), and pulls the phasor distribution away from the NR trajectory. The three-component analysis based on the graphical method developed by Ranjit et al. [32,33] allows for the calculations of NR distribution in the presence of GFP and identifies specific lipid environments for these specific cells (Figure 14h).

## 7. Conclusions

The linear combination properties of phasor plots allow the fractional intensity components of various fluorescent species to be calculated, which can contribute to the fluorescence measured from a single pixel of an image. The relevance of the phasor approach is the ability to perform quantitative analysis of contributions from multiple species without any assumptions about their decay characteristics. We showed that originally the linear combination properties were used to calculate the relative contributions for only two components, but the method has been expanded to three and four components. Additionally, initially these methods involved the knowledge of the phasor positions of the individual components, but recently, they have been developed so they can be used to identify and quantify components without any prior knowledge of their phasor positions using new available electronics. Throughout the article, we have reviewed biological applications for these methods, such as autofluorescence metabolic imaging of NADH and FAD, where calculating the fraction of free or protein-bound can be correlated with changes in cellular metabolism.

Since the start of the phasor approach in the FLIM field, it has expanded to hyperspectral imaging and can be used to obtain quantitative analysis of contributions from multiple spectral components. Multidimensional phasor analysis allows us to combine the analysis for FLIM and hyperspectral imaging (among others). Furthermore, the linear combination has been expanded to MRI imaging to improve the data analysis, including the use of multiple harmonics [58], and also be seen in spectral phasor analysis of hyperspectral Stimulated Raman Scattering (SRS) imaging data [59].

These results show the applicability and the opportunities of using linear combination properties in various phasor-based imaging methods. Our hope is that the linear combination of the phasor space and the newly developed mathematical methods and available electronics will expand the use of the phasor approach in fluorescence imaging.

## Data Availability

No new data were created or analyzed in this study. Data sharing is not applicable to this article.
